# CYFIP1 is directly controlled by NOTCH1 and down-regulated in cutaneous squamous cell carcinoma

**DOI:** 10.1371/journal.pone.0173000

**Published:** 2017-04-14

**Authors:** Piotr J. Dziunycz, Johannes Neu, Karine Lefort, Nadia Djerbi, Sandra N. Freiberger, Guergana Iotzova-Weiss, Lars E. French, Gian-Paolo Dotto, Günther F. Hofbauer

**Affiliations:** 1 Department of Dermatology, University Hospital Zurich, Zurich, Switzerland; 2 Department of Biochemistry, University of Lausanne, Epalinges, Switzerland; University of Alabama at Birmingham, UNITED STATES

## Abstract

Squamous cell carcinoma of the skin (SCC) represents one of the most common cancers in the general population and is associated with a substantial risk of metastasis. Previous work uncovered the functional role of CYFIP1 in epithelial tumors as an invasion inhibitor. It was down-regulated in some cancers and correlated with the metastatic properties of these malignant cells. We investigated its role and expression mechanisms in SCC. We analyzed the expression of CYFIP1 in patient derived SCC, primary keratinocytes and SCC cell lines, and correlated it to the differentiation and NOTCH1 levels. We analyzed the effects of Notch1 manipulation on CYFIP1 expression and confirmed the biding of Notch1 to the CYFIP1 promoter. CYFIP1 expression was down-regulated in SCC and correlated inversely with histological differentiation of tumors. As keratinocyte differentiation depends on Notch1 signaling, we investigated the influence of Notch1 on CYFIP1 expression. CYFIP1 mRNA was highly increased in human Notch1-overexpressing keratinocytes. Further manipulation of the Notch1 pathway in keratinocytes impacted CYFIP1 levels and chromatin immunoprecipitation assay confirmed the direct binding of Notch1 to the CYFIP1 promoter. CYFIP1 may be a link between loss of differentiation and invasive potential in malignant keratinocytes of cutaneous squamous cell carcinoma.

## Introduction

Squamous cell carcinoma of the skin (SCC) belongs to the most common cancers in the world and it is the second most common skin malignancy in the general population [1]. It develops from atypical keratinocytes within sun-damaged epidermis, clinically visible as actinic keratosis or Bowen’s disease, both considered non-invasive forms of SCC [2, 3]. Within the general population, about 1% of affected patients annually develop invasive SCC [4]. Unlike basal cell carcinoma–the most common skin malignancy—cutaneous squamous-cell carcinoma is associated with a substantial risk of metastasis [4]. The overall five-year rate of SCC metastasis is up to 5 percent [5–7]. The risk of recurrence or metastasis is related to the tumor size, location, depth of invasion as well as to histological differentiation [4, 7]. In the study by Rowe et al., poorly differentiated squamous cell carcinomas recurred at a rate of 28.6 percent and the five-year rate of cure after treatment was 61.5 percent, while in contrast well-differentiated tumors had a local-recurrence rate of 1.6 percent with a five-year rate of cure of 94.6 percent. In the study of Schmults et all tumor diameter of at least 2 cm, invasion beyond fat, poor differentiation, perineural invasion, and ear, temple, or anogenital location were risk factors associated with poor outcomes. Other studies have also shown that histological differentiation of tumors strongly correlates inversely with the metastasis rate, where poorly-differentiated SCC behaves most aggressively [8, 9].

Notch signaling is an important form of intracellular communication with a key role in cell-fate determination and differentiation [10]. In keratinocytes it induces differentiation and suppresses tumor development [11]. Its deletion in keratinocytes is sufficient to enhance susceptibility to skin cancer formation [12, 13] and loss of its dermal function contributes to field cancerization with development of intraepithelial and invasive SCC [14]. Notch1 is a trans-membrane receptor that is activated by ligand binding and proteolytic cleavage, with release of the intracellular domain [15]. The activated Notch cytoplasmic domain translocates to the nucleus, where it associates with the DNA-binding protein CSL and an ancillary protein, MamL1 or related family members [16, 17], forming a complex that is required for CSL-dependent transcription. Among others the best characterized targets of Notch1 are HES1, p21 and IRF6 [18–20]. The molecular mechanisms downstream of Notch activation that elicit differentiation remain elusive.

Previous work of Silva et al. described CYFIP1 as a novel putative invasion suppressor in a variety of epithelial cancers [21]. CYFIP1 is a RAC-1-interacting protein [22] which transmits signals from RAC1 to the ARP2/3 complex by modulating the activity of the WASP family members, WAVE1-3, within the WAVE complex. WAVE-mediated activation of ARP2/3 induces the nucleation of G-actin to form a membrane protrusion, called lamellipodium, at the leading edges of cells growing in classical two-dimensional cultures [23–25]. It was shown that Cyfip1 is commonly deleted in epithelial colon, breast or lung cancers. Reduced expression of CYFIP1 was also observed during invasion of these tumors and was associated with a poor prognosis. CYFIP1-mediated depletion of WAVE function reduced epithelial adhesion and led to disorganization of tissue architecture [21].

In the present work, we show that CYFIP1 is a direct Notch1 target in keratinocytes. In this context Notch1 is an indirect inhibitor of cell invasion. These findings are of high clinical significance, as they suggest a rationale for the relationship between squamous cell carcinoma differentiation status and its invasive potential.

## Materials and methods

### Skin SCC samples

Institutional board approval from the Kantonale Ethikkommission Zurich (ethical approval number EK647) for the use of human tissue was granted; all donors signed written informed consent forms in accordance with the Code of Ethics of the World Medical Association (Declaration of Helsinki) for experiments involving humans. All samples were obtained from the University Hospital Zurich (Zurich, Switzerland). Normal skin and SCC samples were obtained from clinical biopsies. Parts not needed for histological diagnosis were processed with institutional board approval. SCC samples (n = 30) were collected for the mRNA expression analyses. The epidermis was mechanically separated from the underlying dermis by a brief heat treatment[26]. Tissues were homogenized in TRIzol reagent (Sigma) for RNA preparation. Immunohistochemistry was performed on tissue microarray composed of 240 invasive SCCs, 46 in-situ SCCs and 11 normal skin samples. The normal skin samples were obtained during abdominoplastic surgery.

### Cell culture

For *in vitro* studies primary keratinocytes were isolated from normal skin obtained from abdominoplastic reductive surgery. 4 mm punch biopsies of healthy skin were kept overnight in CnT-07 Medium (CellnTech), with 1% Diaspase II (Roche, Basel, Switzerland) and 1% antibiotic (Gibco-Initrogen) at 4°C. Keratinocytes were then mechanically separated from dermis and kept in the CnT-07 keratinocyte specific medium to achieve high cell purity. The cell culture was used for experiments until the 5^th^ passage only [19].

Skin SCC13 were obtained from Dotto’s Laboratory (Lausanne University, Epalinges, Switzerland). They were originally reported and subsequently checked (last check in 2008) for the presence of oncogenic mutations [27] (https://cansar.icr.ac.uk/cansar/cell-lines/CAL-27/mutations).

### Plasmids, viruses and siRNA

The Plasmids nNERT-neo and rNeo [28] were kindly provided by Dr. U Just (Christian-Albrechts-University of Kiel, Germany). The pincoNotch1 was obtained by inserting the cDNA of activated Notch1 (from digestion of the pcDNA3/hNIC by BanHI/XhoI) into the BamHi/EcoRI sites of the pincoGFP vector [29]. Primary HKCs or SCC13 cells were transfected with 200 nM of stealth validated two distinct siRNAs (Invitrogen) for human CYFIP1 n°1 (HSS177241) and n°2 (HSS177242).

### Quantitative real-time RT-PCR

Conditions for RNA preparation and RT-PCR were as previously described [13]. The following primers were used: CYFIP1: primer forward 5’- CTGCACGCGGCTCCTTTCCA-3’, primers reverse 5’- GACAAGATGCAGCGGGGCGT -3’; involucrin: primer forward 5’-CACCCGCAGTGTCCAGAGGC-3’, primer reverse 5’-GAGACGGGCCACCTAGCGGA-3’; HES1: primer forward 5’- GGTGCTGATAACAGCGGAAT-3‘, primer reverse 5’- TGAGCAAGTGCTGAGGGTTT-3‘; 36B4 served as an internal control: primer forward: 5’-GCAATGTTGCCAGTGTCTGT-3’, primer reverse 5’- GCCTTGACCTTTTCAGCAAG-3’.

### Immunodetection techniques and antibodies

Conditions for immunoblotting were as described previously [13]. The following antibodies were used: actin (sc-1616), p21 (sc-6246), Notch1 (sc-6014) (Santa Cruz), involucrin (Abcam, ab68) loricrin, filaggrin, Hes1 (AB5702) and CYFIP1 (07–531) (Millipore). Immunohistochemistry was performed as reported previously [30]. Briefly: 3- to 5-μm adjacent sections of formalin-fixed paraffin-embedded tissue arranged in a tissue microarray were used. The deparaffinized sections were heated in a 100-W household microwave oven at maximum power for three times 5 minutes each in 10 mmol/L citric acid for antigen retrieval. Primary antibody was applied for 16 hours at 4°C. Secondary staining was performed using the DAKO APAAP kit. The immunhistochemistry results were quantified by two independent persons. The signal intensity was graded from 1 point which referred to no signal up to 10 points which referred to a very strong signal. The samples were analyzed for the total signal intensity that included the signal from all the epidermis layers.

### ChIP assay

Human epidermis was separated from the underlying dermis by a brief heat treatment and was minced finely in ice-cold PBS. Confluent primary HKCs as well as tissue samples were then cross-linked with 37% formaldehyde to a final concentration of 1% followed by the addition of glycine (final concentration 125 mM). After cross-linking, tissues were washed twice with 10 ml PBS with protease inhibitor. Tissue pellets were processed for ChIP assays as previously described using the rabbit anti-Notch1 antibody (Santa Cruz, C-20) in parallel with affinity-purified non-immune IgGs [20]. Primers used for real-time PCR of the two regions of the human CYFIP1 promoter were: 5’- TAGAGTGTGCTACTATCTGTC-3’ and 5’- GTCTCATCAGATTTCAAAGGG-3’ (-2.9k bp); 5’- ATCCAAAGCCCCTGTTTTGC-3’ and 5’- ATGAAGGTTTGGTTACCCCC-3’ (-0.9k bp). Primers used for a region of human HES1 promoter were: 5’-CCTCCCATTGGCTGAAAGTT-3’ and 5’-CCTGGCGGCCTCTATATATA-3’.

### Luciferase activity assays

Human CYFIP1A promoter was synthetized by Blue Heron Biotech (Bothell, WA) and inserted in pGL3-basic (Promega) between the KpnI and BglII restriction sites (pGL3-CYFIP1A-3kb). SCC13 cells or HKCs were co-transfected with 0.5 μg of pGL3-CYFIP1A-3kb, 2 μg of the Notch1 expressing plasmid pcDNA3-Notch1 or empty vector as control and 0.05 μg of the Renilla internal control plasmid (phRL-TK, Promega). As a control of experiment, cells were also in parallel co transfected with 0.5 μg of the RBPjk-luc plasmid (Notch reporter plasmid), 2 μg of the Notch1 expressing plasmid pcDNA3-Notch1 or empty vector as control (pcDNA3) and 0.05 μg of phRL-TK. Cells were harvested 30 hours after transfection and assayed for Firefly and Renilla luciferase activity with the Dual Luciferase assay reporter kit (Promega). Results are expressed as relative firefly activity over Renilla luciferase activity. All experiments were performed in triplicate.

### Invasion assay

Prior to the assay, cells were incubated for 48 hours at starving conditions with 10 x decreased concentration of supplements. The invasion capacity was assessed using BioCoat Matrigel Invasion Chambers with 8-micron pore size (BD Biosciences, Bedford, MA) following the manufacturer’s protocol. The cells were seeded in the concentration of 1.25x10^5^ cells /ml. As a chemoattractant, full medium was used. After 24 hours, cells were either stained and used for the calculation of invasive capacity or mechanically detached from both sides of the PET membrane and assessed for protein expression by flow cytometry or immunoblotting.

### FACS

Prior to staining, cells were fixed with 1% PFA and permeabilized with 1% saponin. For the assessment of the protein expression the following antibodies were used: unconjugated polyclonal rabbit anti human CYFIP1 (07–531, Millipore) in the dilution 1:100 with FITC conjugated secondary swine anti-rabbit antibody (Dako), diluted 1:25. Measurements were performed on a FACSCanto device (BD Biosciences). Data were analyzed with FlowJo software (Ashland).

### Statistics

All statistical evaluations were carried out using GraphPad Prism 5.0. The analyses were two-tailed Student’s t-test or ANOVA. All real-time RT.PCR samples were tested in triplicates and error bars represent one standard deviation. *P*-values of < 0.05 were considered significant.

## Results

### CYFIP1 is down-regulated in cutaneous SCC

**Fig 1:**
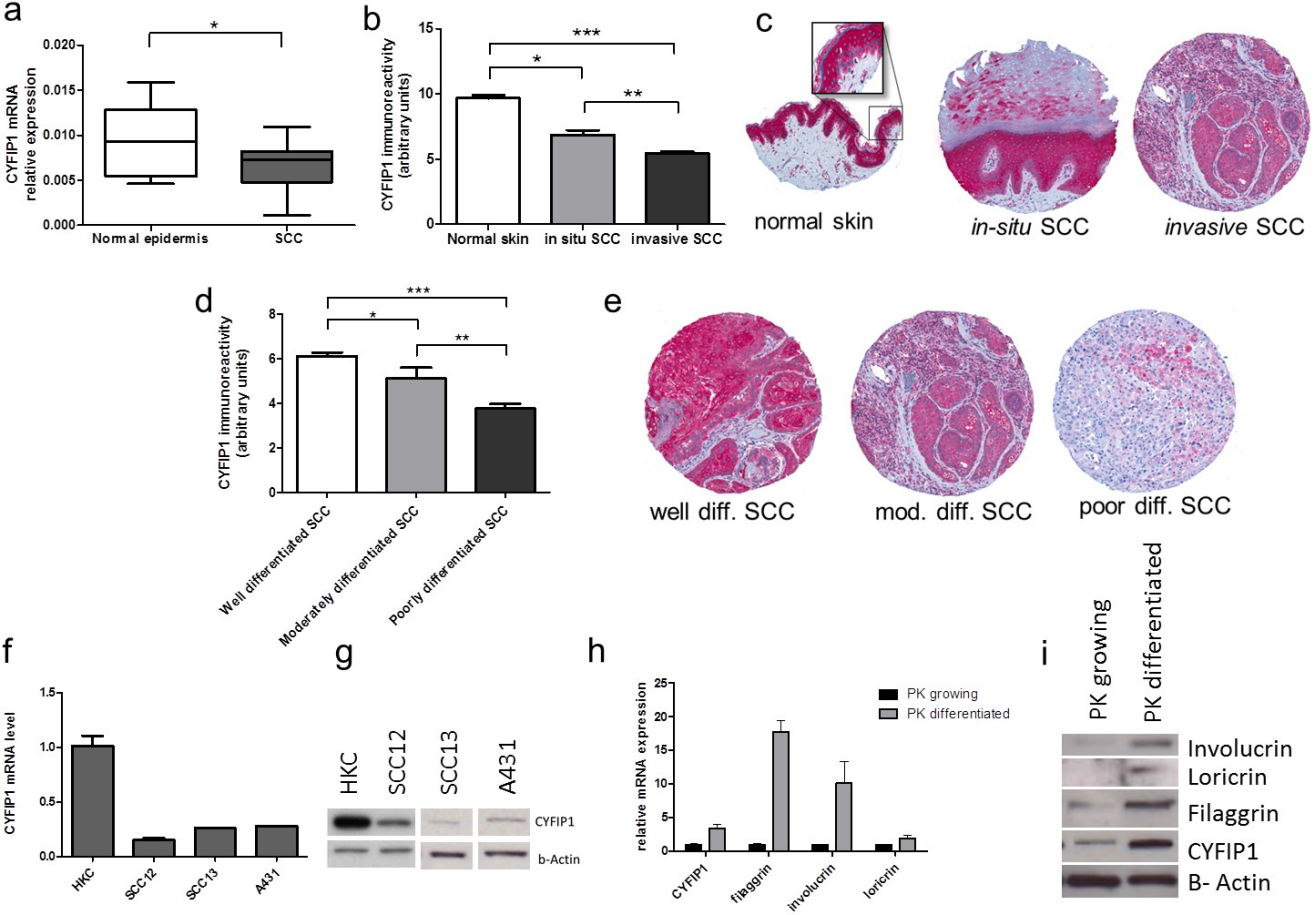
CYFIP1 expression differs between normal keratinocytes and SCC cells. (a) CYFIP1 mRNA expression was measured in epidermis derived from normal skin (n = 9) and from patients SCC (n = 30) samples. The RT-PCR demonstrated significant decrease of CYFIP1 mRNA expression in the tumor samples *p<0.05. (b)CYFIP1 protein expression in clinical samples was detected by immunohistochemistry which was performed on tissue microarray composed of 11 normal skin samples, 46 in-situ SCCs, and 240 invasive SCCs. The analysis revealed significant protein expression differences between all three groups of samples. Keratinocytes in the normal skin show high expression of CYFIP1, which is then decreased in in-situ SCC and even lower in invasive SCC *p<0.05, **p<0.01, ***p<0.001. The representative staining of normal skin, in-situ and invasive SCC are shown in the section c. (d) among the invasive SCC the expression levels of CYFIP1 were related to the histological differentiation of the tumors. Well differentiated SCC showed relatively high expression of CYFIP1, which decreased with the loss of differentiation, so that the lowest expression was observed in poorly differentiated SCC. Fig 1E shows the representative staining of well, moderately and poorly differentiated SCC, *p<0.05, **p<0.01, ***p<0.001. (f) CYFIP1 mRNA expression has been compared between normal human keratinocytes (HKC) and two squamous cell carcinoma cell lines: SCC12, SCC13 and A431. At the time of the experiment the cells were at about 70% of confluence. The CYFIP1 mRNA levels were significantly decreased in the cancer cell lines as compared to normal keratinocytes. (g) Similarly to mRNA levels, CYFIP1 protein levels were significantly lower in the cancer cell lines, as compared to normal human keratinocytes. CYFIP1 expression in normal keratinocytes depends on their differentiation status. CYFIP1 mRNA (h) and protein (i) are expressed in significantly higher amount in differentiated as compared to growing keratinocytes. Cells were at 50% confluence (growing cells), or 100% confluence for 4 days. After this time total RNA and protein were extracted and RT-PCR and WB performed respectively.

Decreased expression of CYFIP1 has been reported in some cancers, such as colon breast or bladder cancer [21].The mRNA expression analysis in epidermis from cutaneous SCC samples showed reduced mRNA expression in tumors compared to the epidermis of normal skin (Fig 1A; normal epidermis: mean 0.011, SD ± 0.0059; SCC: mean 0.0069, SD ± 0.0027; p = 0.034). This difference was further confirmed by immunohistochemistry on 240 samples of SCC samples. CYFIP1 expression was relatively high in normal skin, decreased in in-situ SCC and even more so in invasive tumors (Fig 1B, for representative staining see Fig 1C). Interestingly, CYFIP1 expression was higher in the upper layers of the epidermis, typically layers with advanced differentiation, whereas the cells of the basal layer were mostly negative for CYFIP1. Since these basal cells show the lowest differentiation this finding suggests a relationship between keratinocyte differentiation and CYFIP1 expression.

Further analysis of the invasive SCC demonstrated differential expression of CYFIP1 after stratifying for histological differentiation status. Well-differentiated SCC showed a relatively high CYFIP1 expression, which decreased in parallel with differentiation, i.e. with moderate expression in moderately differentiated tumors and low expression in poorly differentiated tumors (Fig 1D, for representative staining of the well, moderately and poorly differentiated SCCs see Fig 1E).

CYFIP1 expression was next assessed in cultured normal human keratinocytes (HKCs) and in established human squamous cell carcinoma cell lines derived from skin: SCC12, SCC13 and A431. Consistent with the analysis in human SCC samples, SCC12, SCC13 and A431 cell lines demonstrated a similarly decreased expression of CYFIP1 on both mRNA as well as on protein level (Fig 1F and 1G). Consistent with the findings described above, the expression of CYFIP1 as well as of the keratinocyte differentiation markers involucrin, filaggrin and loricrin increased in cultured HKCs upon induction of differentiation by growth to confluence. This up-regulation was observed at both the mRNA and protein levels (Fig 1H and 1I).

### Cyfip1 gene expression is under direct positive Notch1 control in keratinocytes

**Fig 2:**
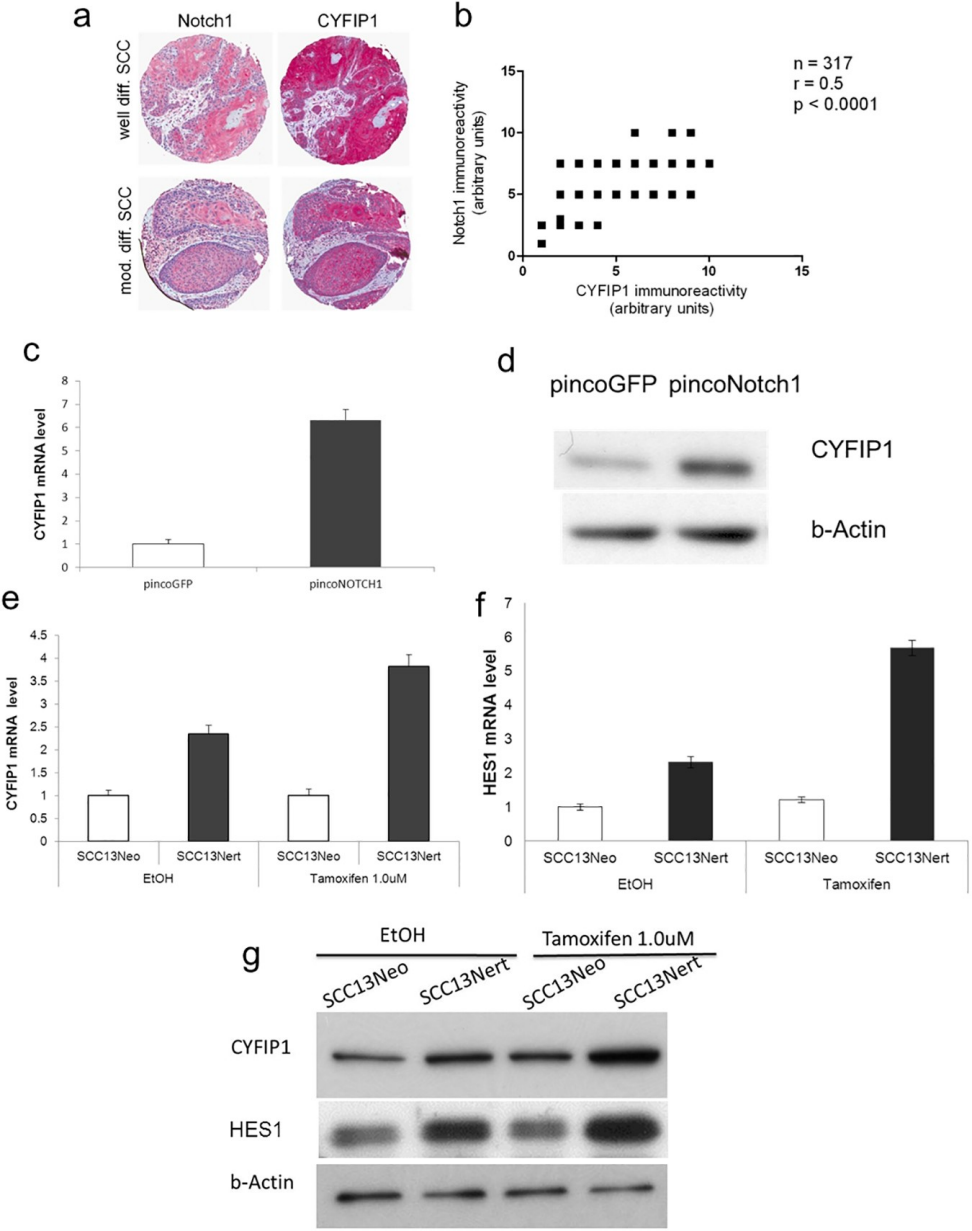
CYFIP1 is induced during keratinocyte differentiation through a Notch-dependent mechanism. (a) 4 um adjacent sections of SCC tissue microarray were separately stained for CYFIP1 and NOTCH1 (n = 286). Immunohistochemical analysis of their expression in human SCC showed similar immunoreactivity patterns for both proteins. The expression was concomitantly increased in the suprabasal epidermis layers. (b) Statistical analysis of the immunoreactivity showed significant correlation of expression between NOTCH1 and CYFIP1 with the r = 0.5; p < 0.0001. (c) SCC13 cells were infected with a recombinant retrovirus expressing constitutively active Notch1 together with GFP (pincoNotch1), or with a virus overexpressing GFP (pincoGFP) alone followed, 72 h later, by mRNA and (d) protein expression analysis. Similar results were obtained in three independent experiments. (e) SCC13 cells were stably infected with a retroviral vector expressing a flag-tagged activated Notch1 protein fused to the human estrogen receptor (SCC13Nert), or empty vector control (SCC13Neo). Cells were subsequently treated with Tamoxifen at 1.0 uM concentration, collected after 30h and analyzed for CYFIP1 and (f) HES1 mRNA and (g) protein expression. Tamoxifen mediated activation of the Notch1 pathway significantly increased the mRNA and protein levels of both CYFIP1 and HES1 which has served as control downstream target.

Notch signaling promotes commitment of keratinocytes towards differentiation and thus prevents development of skin cancer [11]. The immunohistochemical analysis of human SCC showed coincidentally increased expression of the Notch1 and CYFIP1 proteins in upper layers of the epidermis, and differentiated keratinocytes (Fig 2A). The statistical analysis showed a moderate correlation between the immunoreactivity of these two proteins (correlation coefficient r = 0.5; p < 0.0001) (Fig 2B).

To assess whether CYFIP1 expression is under the control of Notch1 signaling pathway, SCC13 cells, previously reported as expressing low levels of Notch1[13], were stably transfected with a retrovirus overexpressing constitutive active form of Notch1. As shown in Fig 2C and 2D, expression of Notch1 led to an induction of CYFIP1 expression on both RNA and protein levels.

These results were further confirmed using SCC13 cells stably transduced with an inducible retroviral vector expressing a flag-tagged activated Notch1 protein fused to the human estrogen receptor (SCC13Nert). Conditional Notch1 expression by 4-hydroxytamoxifen resulted in a substantial induction of CYFIP1 (Fig 2E and 2G) in parallel with HES1 (Fig 2F and 2G), a well-known direct target of Notch1 [31].

**Fig 3:**
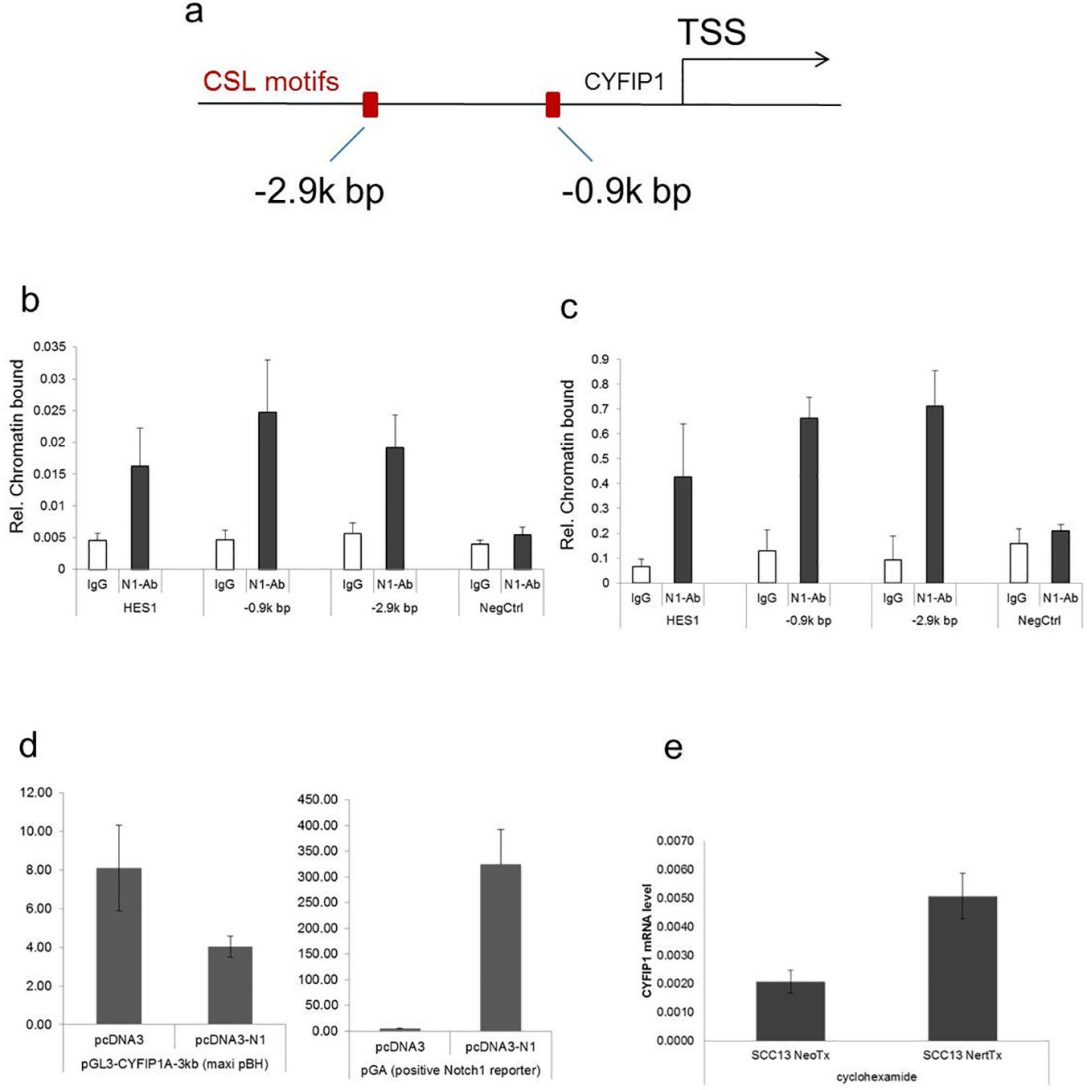
Endogenous Notch1 binds to the CYFIP1 locus within specific regions of chromatin organization. (a) Schematic illustration of ChIP results: TSS–transcription starting site, CLS-binding motifs are represented by the red bars. (b) Human primary keratinocytes and (c) total epidermis extracts of human epidermis were processed for ChIP assays using an antibody specific for Notch1, utilizing non-immune IgGs as control. PCR amplification of the various regions of the human CYFIP1 promoter encompassed the following CSL-binding sites: -2.9k bp: 5’-GAGGTGGGAACTA-3’; -0.9k bp: 5’-AATGTGAGAAAGT-3’. Un-precipitated chromatin preparations were similarly analyzed and used as “input DNA” control. The nucleotide sequence of the PCR primers is given in the materials and methods. The results are representative of two independent experiments. The relative amount of precipitated DNA, expressed in arbitrary units, was calculated after normalization for total input chromatin, according to the following formula (Frank et al. 2001): % total = 2^deltaCt^ x 5 where detaCt = Ct (input)–Ct (immunoprecipitation). Ct, cycle threshold. (d) 3 kb CYFIP1 promotor sequence with the two putative NOTCH1 binding sites was cloned and used for the luciferase activity assay. Increased luciferase activity was observed in response to NOTCH1 overexpression in the positive control (pGA), but not in the cells with the cloned CYFIP1 promotor. (e) To address the question of direct CYFIP1 regulation by Notch1 a further experiment with the protein synthesis inhibitor–cyclohexamide was used. Briefly, NeoNERT cells overexpressing Notch1 protein bound to the estrogen receptor where treated with cycloheximide to block any further protein synthesis. With the addition of tamoxifen, preexisting Notch1 was activated within the NeoNERT cells. This maneuver induced an increase in CYFIP1 mRNA expression, proving the direct nature of the binding of Notch1 to the CYFIP1 promoter.

Further sequence analysis of the proximal region of the human Cyfip1 gene promoter revealed the presence of a “canonical” CSL-binding site located at -2.9kb and -0.9kb from the transcription start site (TSS) (Fig 3A). To verify Notch1 binding to these sites, we performed ChIP assays with extracts from human primary keratinocytes under confluent, differentiating conditions and from normal human epidermis (Fig 3B and 3C respectively). ChiP analysis showed specific binding of the Notch1 protein to both of the predicted motifs within the promoter (-2.9 and -0.9 kb position).

Further functional analysis of the NOTCH1 binding to the Cyfip1 promoter was performed. Interestingly the overexpression of functional Notch1 did not increase the luciferase activity in the construct with the two putative NOTCH1 binding sites of CYFIP1 promoter (Fig 3D). To address the question of the Notch1-Cyfip11 interaction the cyclohexamide protein synthesis inhibition assay was performed (Fig 3E). SCC13 NeoNERT cells overexpressing Notch1 protein bound to the estrogen receptor were treated with cyclohexamide to block any further protein synthesis. With the addition of tamoxifen, preexisting Notch1 was activated within the NeoNert cells. This maneuver induced an increase in CYFIP1 mRNA expression, proving the direct nature of the binding of Notch1 to the CYFIP1 promoter.

### Notch1 regulates cell invasion through CYFIP1

**Fig 4:**
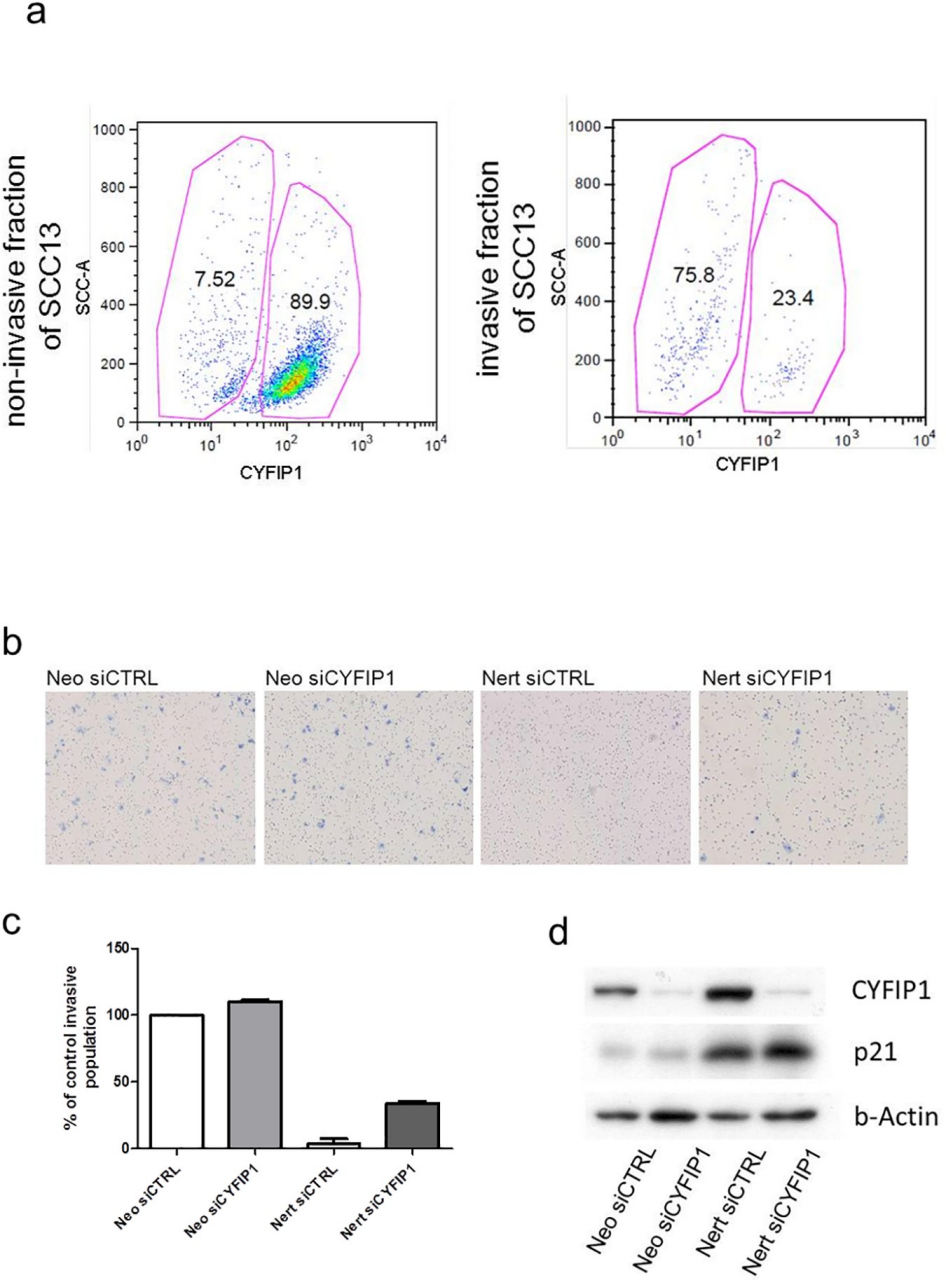
Notch1 inhibits cell invasion through CYFIP1. (a) SCC13 were starved for 2 days and then seeded in the matrigel coated chamber, where the coated membrane with 8 um pores separated the cells from the medium full in supplements. The cells were then cultured for 22 hours. After this timeframe the cells that migrated through the membrane were separated from the remaining cells and thus divided to invasive and non-invasive fraction of SCC13 cells. The cells were next stained for CYFIP1 and analyzed by FACS. The invasive fraction of SCC13 cells shows a lower percentage of CYFIP1 positive cells as compared to the non-invasive fraction (p < 0.001). (b) SCC13 cells were stably infected with a retroviral vector expressing a flag-tagged activated Notch1 protein fused to the human estrogen receptor (SCC13Nert), or empty vector control (SCC13Neo). The cells were subsequently treated with siRNA targeting CYFIP1 mRNA of unspecific control. The cells were next kept in starving conditions, seeded on the matrigel coated membrane with 8 um pores and treated with Tamoxifen. After 22h hours of incubation the invasive and non-invasive cells were counted and the invasion capacity was calculated as indicated in the manufacturer protocol. Section (b) represents the density of the invasive populations in the end of the experiment. (c) SCC13 with activated Notch1 and treated with control siRNA (Nert siCtrl) showed drastically decreased invasive capacity, which could be partially restored when the Notch1 activated SCC13 cells were treated with CYFIP1 specific siRNA (Nert siCYFIP1). (d) For control, the cells were parallel analyzed for CYFIP1 and p21 expression.

To verify the differential expression of CYFIP1 between invasive and non-invasive cells, an invasion assay using a matrigel coated PET membrane was performed. This assay allows the distinction of invasive from non-invasive cells within the population of the SCC13 cell line. Flow cytometry showed a different distribution of CYFIP1-positive versus CYFIP1-negative cells in these two subpopulations (Fig 4A). Within the non-invasive population, 89.9% of the cells brightly expressed CYFIP1, while only 7.52% expressed CYFIP1 dimly. Within the invasive population, however, most cells showed a dim CYFIP1 expression, while only 23.4% brightly expressed CYFIP1 (p < 0.001).

To verify Notch1 as a modulator of invasive capacity, SCC13 cells with Tamoxifen-inducible Notch1 (SCC13Nert) were used in an invasion assay. To assess the interplay between Notch1 activation, Cyfip1 expression and invasion capacity, these cells were treated either with Cyfip1-specific siRNA (NertsiCYFIP1) or control siRNA (NertsiCTRL). Cells stably infected with empty vector control (SCC13Neo) and treated with Cyfip1-specific siRNA (NeosiCYFIP1) or control siRNA (NeosiCTRL) served as control for the Notch1 induction. Interestingly, the induction of Notch1 activity in SCC13 cells dramatically reduced their invasive potential (Fig 4B and 4C) in parallel to the induced expression of Cyfip1 protein (Fig 4D). This reduced invasive phenotype was partially rescued by inhibition of Cyfip1. Induction of Notch1 activity in the SCC13Nert cells was verified by the induction of p21 expression (Fig 4D). This experiment was repeated with two different Cyfip1 specific siRNAs.

## Discussion

The pro-differentiation and tumor suppressive functions of Notch signaling in keratinocytes are well established [11]. However, little is known about its mechanism. It has previously been shown that Notch activation is involved in in the cell-cycle control of keratinocytes via p21^WAF1/Cip1^ [32]. Notch activation also induces differentiation of these cells through a more indirect mechanism, involving modulation of integrin expression in the basal layer, of p63 as well as of IRF family members [19, 20]. We show here that Notch signaling is also involved in the regulation of keratinocyte invasive potential through the modulation of Cyfip1 expression.

To date, CYFIP1 has been shown to inhibit tumor cell invasion in models of colon, lung and breast cancers [21]. It has been demonstrated that in these three particular tumors, its expression is decreased compared to corresponding normal tissues. In our study, Cyfip1 expression was decreased at both the mRNA and protein level in in-situ and invasive SCC when compared to normal skin. Interestingly, progression of in-situ to invasive SCC was associated with a progressive downregulation of CYFIP1 expression, suggesting a function for CYFIP1 as an invasion inhibitor in SCC similar to that reported for other tumors. The histological differentiation of SCC is inversely linked to recurrence, invasion, and metastasis [4, 5, 7–9, 33]. Thus, poorly and undifferentiated SCC have the highest rate of invasion and metastasis, while moderately differentiated SCC were shown to have a higher rate of invasion and metastasis. Our analysis of CYFIP1 expression in well-, moderately- and poorly-differentiated squamous cell carcinomas showed down-regulated CYFIP1 expression in line with a loss of differentiation in tumor cells, suggesting a possible mechanism linking the loss of histological differentiation to increased invasive potential, a phenomenon observed also in other tumors such as esophagus, prostate, colon and breast[34–38].

We show here that Cyfip1 gene transcription is induced in differentiating keratinocytes by a Notch-dependent mechanism. Activation of Notch1 signaling, either directly by overexpression of an active Notch1 variant, or through a conditional manipulation, both increased Cyfip1 mRNA and protein expression in cells. We further confirmed the binding of activated Notch1 to the Cyfip1 promoter within specific CSL-binding sites, revealing Cyfip1 as a direct Notch1 target. This binding was not confirmed by the luciferase assay, but further analysis with the cyclohexamide protein synthesis inhibition assay proved the direct nature of the binding of Notch1 to the Cyfip1 promotor. We also observed decreased levels of Cyfip1 expression in SCC12 and SCC13 cell lines compared to normal human keratinocytes. This decrease can be explained by compromised Notch signaling in these cells [13, 26]. These results explain the observed correlation in the expression of Cyfip1 and Notch1 in the SCC tumors. Both Cyfip1 and Notch1 were relatively weakly expressed in the basal layer of the epidermis, which forms the leading front of the tumors. Both Cyfip1 and Notch1, however, were upregulated in keratinocytes of the spinous and granular layer, e.g. in cells with increasing differentiation.

CYFIP1 expression has been negatively correlated with the invasion of malignant cells in lung, colon, breast and bladder tumors. We assessed whether these phenomena apply to cutaneous SCC as well. To distinguish migration from invasion, SCC13 keratinocytes were cultured on a matrigel-coated membrane. Under the starving conditions and chemotactic stimuli, keratinocytes are forced to inhibit differentiation and move in the direction of the attractant [39]. In line with the reported role of invasion suppressor for CYFIP1, the invasive fraction of SCC13 cells down-regulated their CYFIP1 expression. Our results go along with the results of Silva and colleagues [21] where they also performed an *in vivo* assay where SCC cells with reduced CYFIP1 expression showed drastically increased invasive properties Since Cyfip1 is regulated by Notch signaling and plays the role in cancer cell invasion, the remaining question was if increased Notch signaling may influence this process as well. Our results revealed a drastic decrease in the invasion of SCC cells upon activation of the Notch1 signaling pathway. This occurred in parallel with increased Cyfip1 expression. Interestingly, however, the inhibition of Cyfip1 expression in Notch-activated cells partially rescued the invasive capacity of the SCC cells. These results shed new light on the function of Notch signaling by suggesting Notch1 to function as a promoter of differentiation and an inhibitor of invasion. The Notch1 mediated inhibition of invasion may be partially regulated by the Notch1 mediated induction of CYFIP1 expression. Our data are of likely clinical significance, because they suggest a mechanism for the observed correlation between the loss of tumor differentiation and increased destructive and invasive growth.
